# A Low-Cost Sensor System Installed in Buses to Monitor Air Quality in Cities

**DOI:** 10.3390/ijerph20054073

**Published:** 2023-02-24

**Authors:** Carolina Correia, Vânia Martins, Bernardo Matroca, Pedro Santana, Pedro Mariano, Alexandre Almeida, Susana Marta Almeida

**Affiliations:** 1 Centro de Ciências e Tecnologias Nucleares, Instituto Superior Técnico, Universidade de Lisboa, Estrada Nacional 10, 2695-066 Bobadela, Portugal; 2 ISCTE—Instituto Universitário de Lisboa (ISCTE-IUL), Av. das Forças Armadas, 1649-026 Lisboa, Portugal; 3 ISTAR—Information Sciences and Technologies and Architecture Research Center, Av. das Forças Armadas, 1649-026 Lisboa, Portugal; 4 Instituto de Telecomunicações, Av. Rovisco Pais, 1, 1049-001 Lisboa, Portugal

**Keywords:** air quality, low-cost sensors, particulate matter, mobile experiments, calibration

## Abstract

Air pollution is an important source of morbidity and mortality. It is essential to understand to what levels of air pollution citizens are exposed, especially in urban areas. Low-cost sensors are an easy-to-use option to obtain real-time air quality (AQ) data, provided that they go through specific quality control procedures. This paper evaluates the reliability of the ExpoLIS system. This system is composed of sensor nodes installed in buses, and a Health Optimal Routing Service App to inform the commuters about their exposure, dose, and the transport’s emissions. A sensor node, including a particulate matter (PM) sensor (Alphasense OPC-N3), was evaluated in laboratory conditions and at an AQ monitoring station. In laboratory conditions (approximately constant temperature and humidity conditions), the PM sensor obtained excellent correlations (R^2^≈1) against the reference equipment. At the monitoring station, the OPC-N3 showed considerable data dispersion. After several corrections based on the k-Köhler theory and Multiple Regression Analysis, the deviation was reduced and the correlation with the reference improved. Finally, the ExpoLIS system was installed, leading to the production of AQ maps with high spatial and temporal resolution, and to the demonstration of the Health Optimal Routing Service App as a valuable tool.

## 1. Introduction

Air pollution represents one of the biggest environmental risks to human health leading to increased morbidity and mortality due to cardiovascular and respiratory diseases [1,2,3]. While several measures have already been implemented in urban areas to tackle air quality (AQ) deterioration such as the improvement of public transportation [4,5,6], the levels of pollutants to which the citizens are exposed are still high and above the guidelines defined by the World Health Organization (WHO). The road transport sector is one of the major contributors to air pollution in urban areas [7]. In fact, in the transports microenvironment, commuters are particularly exposed to high levels of air pollution [8,9,10].

In urban areas, air pollutant monitoring and exposure assessment studies are traditionally performed through complex stationary equipment at fixed monitoring stations. Although very reliable, these stations are only able to characterize AQ in their vicinity and, due to the high acquisition and maintenance costs, they are installed in a small number of points within cities. Consequently, the spatial characterization of air pollutants’ concentrations in urban areas is not fully achieved, compromising a correct assessment of the population’s exposure to atmospheric pollutants [11], which can be accomplished through, for example, location-specific analysis [12].

Nowadays, low-cost sensors (LCS) are an increasingly attractive option to complement the data produced by the reference equipment from fixed monitoring stations [13]. These AQ sensors are low-cost, small, easy-to-use, and portable, which allows for the generation of massive quantities of information. Thus, they have been playing an important role in monitoring pollution trends, finding hot spots, identifying pollution sources, informing the population, and supporting environmental management [14,15,16,17].

However, although they have the capability to produce high temporally and spatially resolved AQ data, they tend to have more severe data quality problems compared to the conventional AQ monitoring instruments. To guarantee the quality of the data produced, LCS need to be calibrated and corrected against several factors. The complexity of particulate matter (PM) due to its emission sources, chemical composition, particle sizes, concentration ranges, and the environments in which the sensors are used led to the development of different calibration methods. The calibration of LCS may be developed at a laboratory or on-field. Laboratory caalibration is often seen as the most appropriate method since it makes it possible to exploit how different factors influence the sensors’ response. On the other hand, on-field calibration can be the most appropriate method as it is carried out under the conditions in which the sensors will be used, although without the opportunity to analyze to what extent different factors influence the sensors’ measurements. In both cases, calibration is usually performed by placing an LCS next to reference equipment.

PM LCS’ readings are mostly influenced by meteorological factors, such as temperature (T) or relative humidity (RH) [15,16,17,18,19,20]. The most common methods to account for this effect in the calibration process include linear and multilinear regression analysis and specific RH correction techniques, which are further investigated in Section 2.3. In addition to this method, machine learning techniques [21,22,23,24,25,26] can also be employed to correct the sensors’ data, having the advantage of including other factors in its analysis, such as the period of the day and characteristics of the area where the data is collected. While this may be seen as an advantage, it could transform the calibration process into a very location and condition-specific process, failing to generalize the calibration procedure [26]. In this paper, the LCS’ calibration considers only the correlation with reference equipment and the influence of meteorological factors, as they are recognized as some of the main factors influencing the sensors’ performance.

The ExpoLIS project developed a low-cost sensing system to be installed on the top of public buses which can cover an entire city generating a massive quantity of data with high spatial and temporal resolution, and a Health Optimal Routing Service App that aims to expand the citizens’ knowledge about AQ and to reduce their exposure during commuting. This study aims to evaluate the performance of these ExpoLIS tools to measure PM concentrations in several conditions.

## 2. Methods

### 2.1. ExpoLIS LCS Sensor Node Prototype

**Figure 1:**
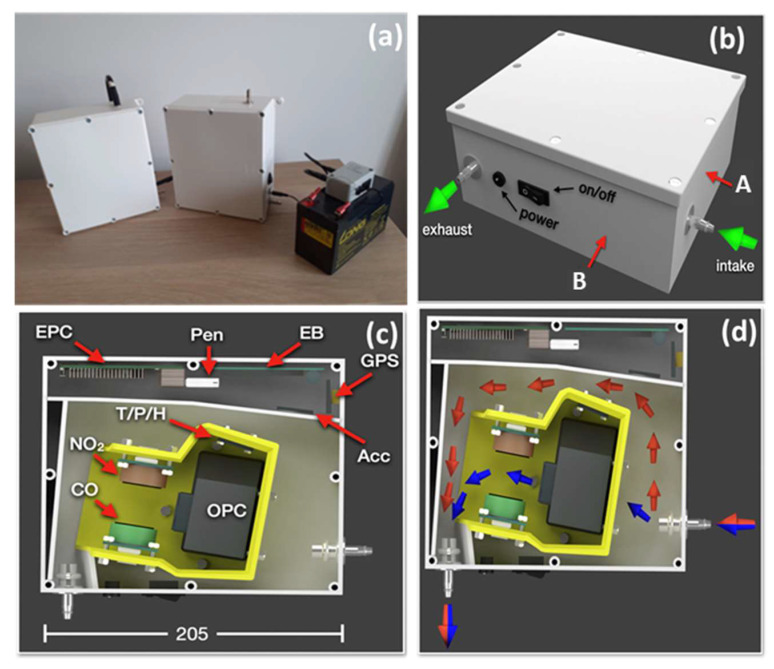
Sensor node details: (**a**) ExpoLIS sensor node prototype; (**b**) sensor node 3D model, including the representation of the air inlet (A) and the exhaust (B) surfaces; (**c**) LCSs and electronic devices included in the sensor box (EPC: Embedded Computer, Pen: USB Drive, EB: Custom Electronics Board, GPS: Global Positioning System device, Acc: Accelerometer, T/P/H: T, P and RH sensors, OPC: OPC-N3 sensor, NO_2_: NO2-A43F sensor and CO: CO-B4 sensor) and (**d**) air flow in the interior of the sensor node.

The ExpoLIS sensor node prototype (for more details about the Sensor Node prototype, including the code and the model for 3d printing, see https://github.com/ExpoLIS-project—accessed on 23 February 2022) (Figure 1a) resulted from several trials that intended to improve its robustness and the accuracy of the installed sensors [27]. It is composed of an air inlet (in surface A in Figure 1b) to which can be connected a conductive tube to extend the air intake point. To ensure that an adequate quantity of air passes through the sensors without any delays, the exhaust point (in surface B in Figure 1b) is prepared to be connected to a conventional air pump to create an adequate airflow, preferably at a rate of 9 L min^−1^ (a Leland Legacy air pump from SKC Inc. was used in this study). The sensor node’s power supply can fluctuate between 12 volts of direct current (VDC) and 24 VDC, which can be provided either by a car lighter, a battery pack, or an AC/DC converter connected to an electrical outlet.

In the interior of the sensor node, the allocation of each component reflects the need to ensure the smallest occupied space possible and an adequate airflow through the AQ LCS. The sensor node hardware is divided in two different categories: (1) the LCS and (2) the electronic devices, as represented in Figure 1c.

The sensor node is equipped with three AQ sensors and a meteorological sensor. A miniaturized optical particle counter (Model OPC-N3 from Alphasense) employs the principle of light scattering to count the numerical concentrations of PM that reach the detection chamber. These concentrations are converted on board to mass concentrations according to the European standard EN 481. Although the sensor provides concentrations of several size bins, for this study we only considered the concentrations of PM with aerodynamic diameters smaller than 2.5 µm (PM2.5) and 10 µm (PM10). Although this work only focuses on the measurement of PM, two electrochemical four-electrode gas sensors from Alphasense were also installed to monitor the concentrations of CO (CO-B4, Alphasense) and NO_2_ (NO2-A43F, Alphasense). These sensors’ working principle is based on the reaction of the given gas with a specific electrolyte that generates an electrical current. The sensors’ output is composed of two different voltages, one from a working electrode and one from an auxiliary electrode, whose difference is proportional to the target gas concentrations. These voltages are periodically sampled by an analog-to-digital converter (ADC). Regarding the meteorological conditions, the sensor node monitors T, RH (SHTC3, Adafruit), and the barometric pressure (P) (LPS25, Adafruit). Each one of the measured concentrations is delivered at the rate of one second to accurately monitor concentrations while in motion.

As the electronic devices need to be protected against water droplets or moisture, they are placed in a section separated from the air flow (Figure 1c). Power distribution and sensor interfacing are handled by a custom electronics board. This board includes 16-bit analog-to-digital converters (ADS1115, Adafruit) for interfacing the gas sensors, and an accelerometer (MSA 301, Adafruit) for registering the sensor node’s vibration and tilt angles. A GPS device (PA1010D, Adafruit) that geo references the data is fixed to one of the interior surfaces of the sensor node to ensure a proper sky view. Finally, the electronics board is connected to an embedded computer, RaspberryPI 3B+, responsible for processing and storing the gathered sensor data in an USB drive.

The sensor node’s configuration allows the air that enters to circulate in the area allocated to the AQ LCS through the OPC-N3 that already has an integrated fan promoting airflow (blue arrows in Figure 1d). To avoid overflowing the OPC-N3 with any remaining air, a secondary path allows this air to easily surround the yellow 3D printed object and exit the sensor node at the exhaust point (red arrows in Figure 1d).

### 2.2. Prototype Quality Control

#### 2.2.1. Laboratory Tests

The prototype’s quality control, and subsequent calibration, was performed through two laboratory tests to evaluate how specific factors can influence the data produced by the LCS. The first test was performed in a laboratory for 39 h and aimed at evaluating the performance of the sensors under controlled T and RH conditions. The LCS were exposed to an air pollution source (incense stick) to evaluate their response to several PM concentrations. Secondly, the sensors were placed in a garage for 14 h, where they were exposed to vehicle exhaust and higher variations of T and RH. In these tests, measurements were made in parallel with a light-scattering laser photometer (DustTrak, Model 8533, TSI Inc. Shoreview, MN, USA). This equipment is an optical instrument that measures simultaneously the mass concentrations of particles with several aerodynamic diameters, namely PM2.5 and PM10. The DustTrak measurements were corrected against reference PM concentration data obtained by gravimetry using a sampler (MVS6, Sven Leckel, Germany), which is certified according to CEN EN 12341. This sampler collected PM2.5 in Teflon filters and PM2.5-10 in nucleopore filters during 24 h periods at a constant flow rate of 2.3 m^3^h^−1^ (following the same approach as in [28]), which were weighted before and after sampling in a microbalance (R160P, Sartorius, Germany).

#### 2.2.2. Field Tests

Ambient environment is complex, so laboratory calibration may not be sufficient to correct for the outdoor conditions to which the LCS are exposed. Thus, it is necessary to perform field calibrations that take into account meteorological factors that can affect the LCS’ performance [29,30]. The outdoor evaluation of the ExpoLIS sensor node system performance was accomplished in one of the Lisbon urban background AQ monitoring stations (Olivais station) from the Portuguese AQ Monitoring Network. The station is equipped with reference instruments that measure PM2.5 and PM10 concentrations using beta attenuation technology (Environment MP101M, Envea, France). At this location, PM concentrations measured by the ExpoLIS sensor node system were compared with concentrations provided by the station’s reference equipment and the DustTrak 8533 for 14 days.

### 2.3. Correction of the Factors Affecting PM Concentrations

LCS’ PM readings are known to be affected by meteorological conditions, thus requiring a correction over the original data. A straightforward correction of the data against reference equipment can be achieved through a simple linear regression (SLR). On the other hand, to correct the influence of T and RH, two approaches can be considered: the application of a multiple linear regression (MLR), and the use of the k-Köhler theory to correct for the effect of the particles’ hygroscopicity.

In this study, SLR and MLR were used to correct the OPC-N3 data against the reference equipment without accounting for T and RH (Equation (1)), as well as to consider the effect of T (Equation (2)), RH (Equation (3)), and of both meteorological parameters (Equation (4)):

(1) $$PM_{OPC-N3}=a\times PM_{reference}+b,$$

(2) $$PM_{OPC-N3}=c\times PM_{reference}+d\times T+e,$$

(3) $$PM_{OPC-N3}=f\times PM_{reference}+g\times RH+h,$$

(4) $$PM_{OPC-N3}=i\times PM_{reference}+j\times T+k\times RH+l$$

where *PM_OPC-N3_* are the concentrations measured by the OPC-N3, and *PM_reference_* the concentrations measured by the reference equipment, both in µg m**^−^**^3^.

During periods of high levels of RH, PM concentration readings may be incorrectly higher due to the ability of particles to absorb water [15,19]. The k-Köhler theory relates the particles hygroscopicity and their volume, as presented in Equation (5) [15,20]:

(5) $$\frac{m}{m_{0}}=1+\frac{\frac{\rho_{w}}{\rho_{p}}\;k}{-1+\frac{1}{a_{w}}},$$

where $\frac{m}{m_{0}}$ represents the ratio between the wet *(*m*)* and dry *(*$m_{0}$*)* particles’ mass, *ρ_w_* and *ρ_p_* are the water density (1 g cm**^−^**^3^) and particle density (assumed to be 1.65 g cm**^−^**^3^), respectively; k represents the slope of the exponential line of the humidogram and, finally, *a_w_* is the water activity (determined as 1/RH).

In this study, the wet particle mass corresponds to the OPC-N3 readings and the dry particle mass to the Environment MP101M instrument, as it is equipped with an air dryer that removes humidity from the entering air.

### 2.4. Prototype Quality Control

Finally, four ExpoLIS sensor nodes were installed in four public buses to test their ability to measure AQ during movement. In each measurement route, concentrations were measured inside and outside of buses; being that these data was also used as an input to the ExpoLIS Health Optimal Routing Service App. This app was designed to show AQ maps produced by the sensors installed in the buses and to help citizens to reduce their exposure to air pollutants while commuting [31].

The Municipality of Lisbon defined a system of hierarchical levels to characterize the road networks that consists of four different levels [32]:

- Level 1: Structuring road network, which supports long distance routes;
- Level 2: Main distribution network, whose function is to distribute traffic between the different sectors of the city;
- Level 3: A secondary distribution network that supports the proximity distribution;
- Level 4: A network of proximity that works at the levels of collection and distribution within neighborhoods and as local accesses.

**Figure 2:**
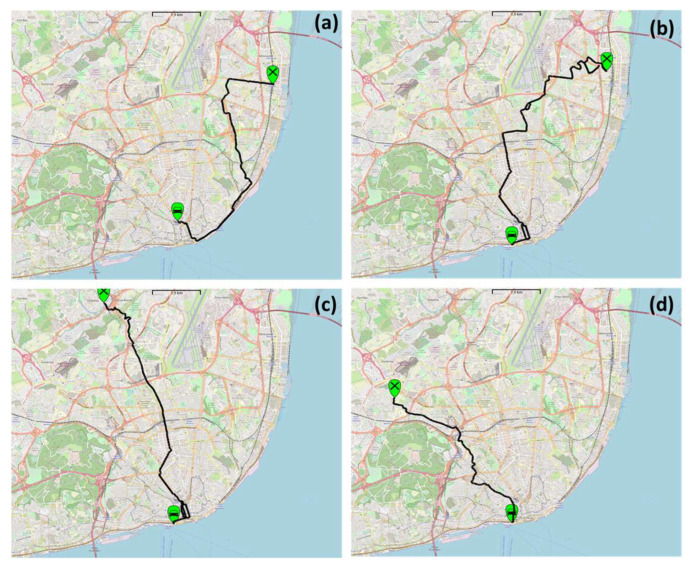
Bus routes selected for the measurements campaign: (**a**) Restauradores—Oriente; (**b**) Restauradores—Moscavide; (**c**) Odivelas—Cais do Sodré and (**d**) Portas de Benfica—Cais do Sodré.

Following this approach, four routes were selected for the measurement campaign (Figure 2) considering the need to obtain diverse routes with different characteristics, as presented in Table 1.

## 3. Results

### 3.1. Intercomparison between Reference Instruments

The DustTrak 8533 and the Environment MP101M were used as reference equipment because they provide good time resolution, fast response signal, excellent signal-to-noise ratio, and simplicity. The data provided by these instruments was firstly corrected against simultaneous gravimetric PM2.5 and PM10 measurements. 

**Figure 3:**
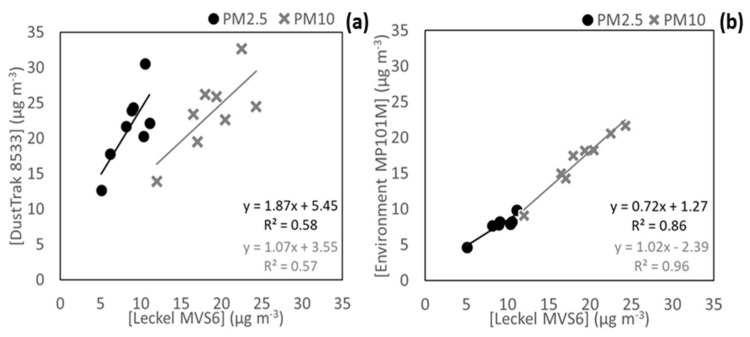
(**a**) Correlation between the gravimetric PM mass concentrations and the DustTrak 8533 and (**b**) the Environment MP101M measurements.

Figure 3 displays the comparison of PM2.5 and PM10 concentrations determined gravimetrically (by a Leckel MVS6) against those measured by the DustTrak 8533 and the Environment MP101M, which were averaged in 24 h periods. A low correlation between the DustTrak 8533 and Leckel measurements was obtained, and therefore PM2.5 and PM10 concentrations measured during this work were corrected based on the linear regression equations shown in Figure 3a. On the contrary, a good correlation (Figure 3b) was obtained in the intercomparison between the Environment MP101M and Leckel data (R^2^ = 0.86 for PM2.5 and R^2^ = 0.96 for PM10). 

### 3.2. Quality Control of the ExpoLIS Sensor Node in Laboratory Tests

The ExpoLIS sensor nodes were firstly tested in laboratory with small variations of T (27.9*–*28.9 °C) and RH (45.4*–*48.4%) values against the DustTrak 8533. The LCS sensor nodes were tested under steadily increasing concentrations, which varied between 0.3 and 38.0 μg m^–3^ for PM2.5, and between 0.3 and 51.7 μg m^–3^ for PM10 (as measured by the OPC-N3).

**Figure 4:**
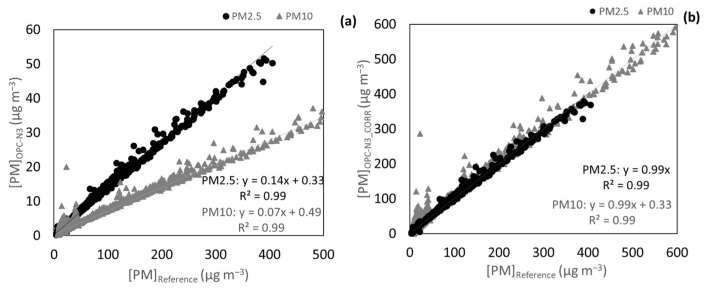
(**a**) SLR of the concentrations measured by the OPC-N3 and DustTrak 8533 for PM2.5 and PM10 and (**b**) consequent correction. Measurements done in a laboratory setting.

The OPC-N3 and the DustTrak had an excellent correlation (R^2^ equal to 0.99). However, the OPC-N3 underestimated the concentrations indicating the need to correct the measured concentrations based on the SLR equation presented in Figure 4a. As indicated in Figure 4b, the underestimation of the sensors’ reading was solved with this approach, while keeping the correlation between the two pieces of equipment.

**Figure 5:**
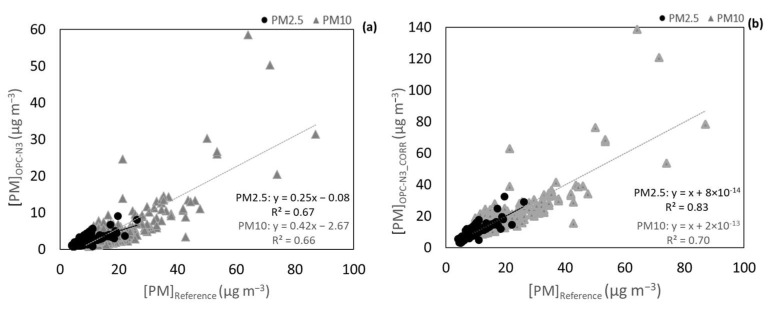
(**a**) Correlation between the OPC-N3 PM2.5 and PM10 concentrations and the correspondent concentrations measured by the DustTrak and (**b**) correlation between OPC-N3 PM2.5 and PM10 concentrations corrected with a MLR considering T and HR and concentrations measured by the DustTrak. Measurements done in an indoor environment with a vehicle exhaust as a pollution emission source.

Furthermore, tests were performed in a garage where the ExpoLIS sensor nodes were exposed to vehicle exhaust and a higher variation of T (26.5*–*31.8 °C) and RH (28.2*–*41.0%). Figure 5 shows that the agreement between the OPC-N3 and the DustTrak got worse, possibly because of the lower range of concentrations inside the garage (from 0.7 to 9.1 μg m*^−^*^3^ for PM2.5 and from 0.8 to 58.5 μg m*^−^*^3^ for PM10 concentrations, as measured by the OPC-N3), considering that, as it has already been seen, the correlation tends to be higher for larger ranges of concentrations. In addition, the variation of T and RH was higher than in the previous test, and therefore, data was corrected with MLR considering RH and T according Equation (5). Figure 5b shows that with a correction considering the effect of T and RH, the agreement improved and the underestimation of concentrations by the OPC-N3 was corrected.

### 3.3. Quality Control of the ExpoLIS Sensor Node in Field Tests

While the results from laboratory measurements are a good performance indicator, they are not representative of the actual conditions in which the sensors will be measuring air pollutants concentrations. There are several factors, including meteorological conditions (T and RH), which influence the sensors performance and their ability to measure PM concentrations. Therefore, the ExpoLIS sensor node system was installed at the Olivais AQ Monitoring Station where it measured concentrations in parallel with the equipment Environment MP101M and DustTrak 8533. 

**Figure 6:**
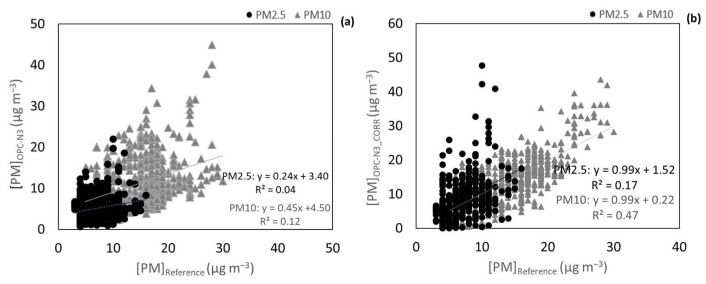
(**a**) Correlation of PM2.5 and PM10 OPC-N3 concentrations data against the Environment MP101M (**b**) corrected PM2.5 and PM10 concentrations. Measurements done at an AQ monitoring station.

For the OPC-N3, the concentration range of PM2.5 was from 0.7 to 21.9 μg m*^−^*^3^, and for PM10 it varied between 1.6 and 44.9 μg m*^−^*^3^ during a test period of 14 days. The results from the comparison between the OPC-N3 and the Environment MP101M were not satisfactory. Figure 6a shows weak correlations (R^2^ equal to 0.04 and 0.12 for PM2.5 and PM10, respectively) and a high dispersion of the values, which has already been reported in other studies that evaluated the performance of this sensor in real-world conditions [33].

During the tests, a high variation of RH (21.9–89.2%) was observed. In addition, it was possible to verify that high RHs were usually associated with high OPC-N3 readings, especially for PM10 (Figure S1). The k-Köhler theory was applied to correct the effect of RH. For its application, the data from the Environment MP101M was considered as the mass of dry particles, and the OPC-N3 concentrations data the mass of wet particles, through the principles of Equation (5). After the correction, the correlation coefficients of PM10 improved with R^2^ values increasing from 0.12 to 0.47. In addition, the slopes’ values improved from 0.45 to 0.99, which indicates that the PM10 concentrations measured by the sensors were in more agreement with the values reported by the reference.

For PM2.5, although there was an effect of RH on the measured concentrations, there was not an evident exponential tendency (Figure S2), which is a criterion for the application of the k-Köhler theory. Therefore, the correction, which considered the effects of RH and T, was applied using a MLR analysis. As a result, there was an improvement in both the values of R^2^ (from 0.04 to 0.17) and in the slopes of the correlation line (from 0.25 to 0.99). As it also happened for PM10, the corrections for PM2.5 and PM10 successfully improved the measured data, in spite of the dispersion of the data that was not totally solved.

### 3.4. Deployment of the ExpoLIS Exposure System in Lisbon

After the evaluation of the sensor node performance and the implementation of the correction obtained in the field tests, the ExpoLIS system was deployed in Lisbon to assess the behavior of a sensor node in the field, to produce concentration maps, and to test all the functionalities of the ExpoLIS App (for more details regarding the ExpoLIS App see https://github.com/ExpoLIS-project/expolis-mobile-app—accessed on 23 February 2022) [27]. In each bus route, two sensor nodes were installed to simultaneously measure the indoor and outdoor PM concentrations, and measurements were made twice a day, at 8 a.m. and at 8 p.m. Table 2 summarizes the average PM concentrations measured by the sensor node along the four selected bus routes and the corresponding meteorological conditions. The indoor average concentrations were 6.9 µg m^−3^ for PM2.5 and 27.7 µg m^−3^ for PM10. However, the concentrations to which the passengers were exposed (PM Indoor concentrations) varied according to the route and starting time. In general, PM2.5 indoor concentrations were higher than the concentrations measured outdoors (4.1 µg m^−3^ for PM2.5 and 15.0 µg m^−3^ for PM10) with I/O ratios varying between 0.9 and 3.3 for PM2.5, and between 1.2 and 5.0 for PM10.

**Figure 7:**
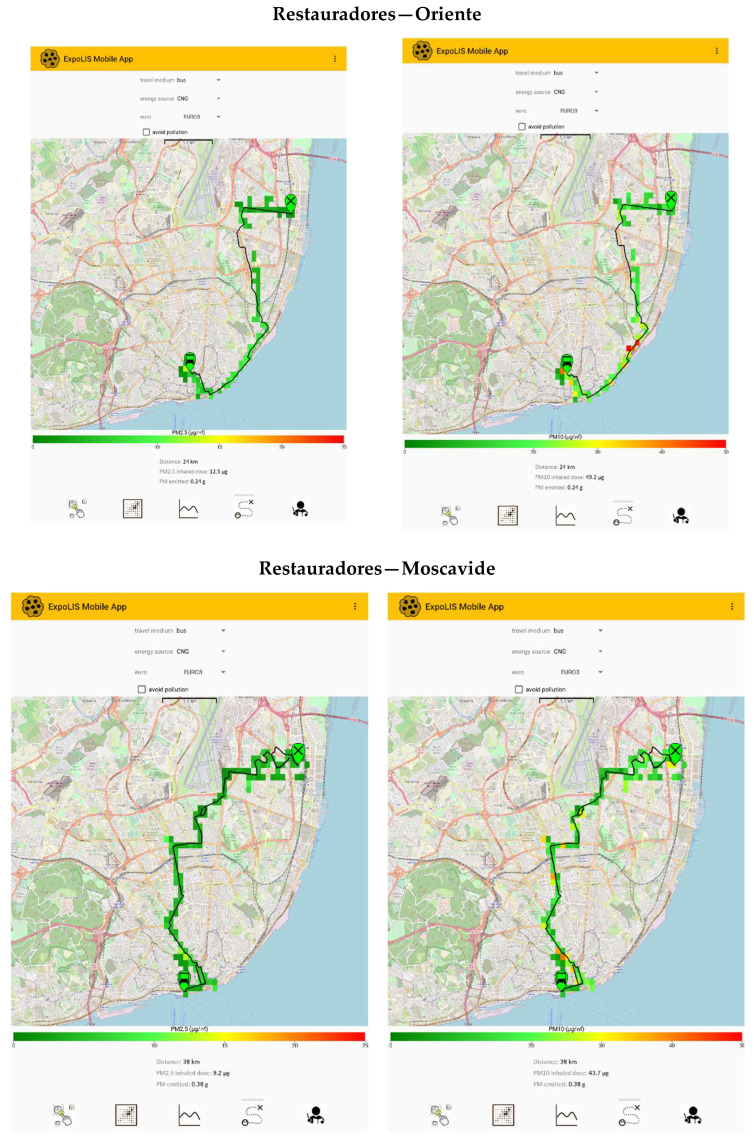

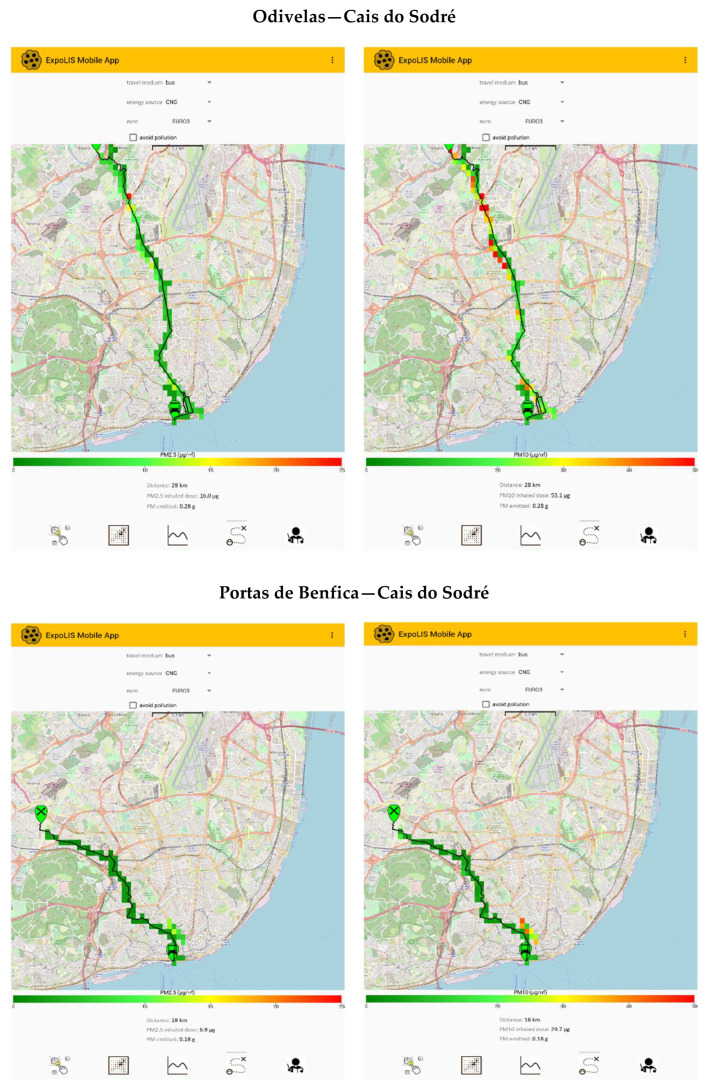
Screenshots of the ExpoLIS Health Optimal Routing Service App for the four selected routes when commuting by a Euro 3 bus powered by Compressed Natural Gas (CNG), with the representation of the spatial distribution of outdoor PM2.5 and PM10 concentrations, and the indication of the respective PM inhaled dose and emission.

The results show that the high PM concentrations to which the citizens are exposed during commuting can lead to a substantial contribution to their total daily exposure and inhalation of air pollutants, especially in high vehicle-density metropolitan areas. Figure 7 shows the ability of the ExpoLIS Health Optimal Routing Service App to provide to Lisbon’s citizens not only the spatial distribution of outdoor PM concentrations along a selected route, but also the total PM inhaled dose and emission. Moreover, this app provides a routing service in which the users can select their starting and ending points, as well as the option “avoid pollution”, receiving in turn the route that minimizes exposure to air pollutants. The App allows the user to compare (i) the dose of pollutants inhaled during commuting in different transport modes (i.e.*,* Bus, Car, Motorcycle, Bicycle and Walking) and (ii) the pollutants’ emission of the motorized modes, considering not only the type of fuel but also the Euro standard.

#### 3.4.1. Reducing the PM Inhaled Dose through the Use of the ExpoLIS App

In the ExpoLIS App the PM inhaled dose (*D_PM_* in µg) along a selected route is estimated using Equation (6):

(6) $$D_{PM}=PM_{out\;}\times \frac{I}{O}\times t\times IR,$$

where *PM_out_* is the average outdoor PM concentration (µg m^−3^), I/O the indoor-to-outdoor PM ratio, t the travel time (h) and IR the inhalation rate (m^3^ h^−1^). Although in this work the indoor and outdoor concentrations in buses were measured simultaneously (Section 2.4), the deployment of the system in cities only included the installation of the sensors outdoors. Therefore, in the App, the concentrations to which the citizens are exposed during commuting are derived from the outdoor concentrations through the application of the I/O ratios. The PM I/O ratios generated in this work for buses, and in a previous study [8] for cars, were a valuable base information for the development of the ExpoLIS App. The I/O values considered were 2.10 and 2.50 in buses and 0.96 and 0.93 in cars, for PM2.5 and PM10, respectively. These values should be updated in the App whenever more scientific knowledge is available.

For motorcycle, bicycle, and walking, the I/O ratio used was equal to 1. The inhalation rates are based on two studies developed previously [34,35]. Table 3 displays the IR used for the different commuting modes.

Table 4 presents the PM2.5 and PM10 inhaled doses reported by the ExpoLIS App for each selected route, for different transport modes and considering a two-way trip. The highest PM2.5 and PM10 inhaled doses were obtained when travelling by bus and on bicycles, due to the high indoor PM concentrations and inhalation rate, respectively. In buses, the high indoor PM concentrations are mainly associated with the resuspension of particles, created by the passengers’ movement and the air flowing in and out when the doors are opened [36]. Moreover, bus passengers are exposed to high pollution at stops when the doors are opened, often in places where queues of idling vehicles are releasing high levels of air pollutants. 

#### 3.4.2. Reduction of the PM Exhaust Emissions through the Use of the ExpoLIS App

The ExpoLIS App provides the exhaust PM emissions of the motorized modes, considering not only the type of fuel, but also the Euro standard. The pollutant emission per passenger, (*E*; g of pollutant emitted per passenger) is calculated by the product between the emission factor (*EF*; g km^−1^) for each motorized transport mode and the total distance of the route (*d*; km) for a two-way trip divided by the number of passengers (p), considering one passenger for cars and motorcycles and fifty passengers for buses (Equation (7)):

(7) $$E=\frac{EF\times d}{p},$$

The emission factors of the European Monitoring and Evaluation Programme (EMEP) air pollutant emission inventory guidebook published by the European Environment Agency [37] were used in the App calculations.

In this work, using the emission factors presented in Table 5, we compared PM emissions for different vehicles, fuel type, and Euro standard as indicated in Table 6. The highest number of particles was emitted by buses, followed by motorcycles and cars. Euro 5 diesel buses produced 126 times more particles per kilometer than Euro 5 diesel cars, but they typically carry around 50 times more people. Thus, although traveling by car showed the lowest PM2.5 and PM10 inhaled doses among the motorized transport modes, it presented similar emissions per passenger when commuting by bus (depending on the type of fuel considered), considering that 50 people are at each bus. Results also show that in cities, the selection of the bus fleet regarding the type of fuel is crucial for the air quality. The Diesel buses have emissions three times higher than the CNG buses. As motorcycles do not have emission control devices like catalytic converters to neutralize the released pollutants, in general they emit more PM than cars.

## 4. Conclusions

This study presented the ExpoLIS system which consists of a network of LCS that monitor AQ, and a Health Optimal Routing Service App. In this paper, the authors intended to perform a preliminary evaluation of the ExpoLIS sensor node and application of the Health Optimal Routing Service App. 

Under controlled conditions in a lab, the PM LCS had an excellent performance, requiring only a simple correction (through SLR) to overcome the LCS’ underestimation of concentrations. However, when exposed to a higher variation of T and RH, the performance of the LCS demonstrated the need to correct the measured concentrations considering these meteorological factors.

In the field test, an exponential increase of the PM10 concentrations with the increase of the RH was observed, and therefore a correction was performed based on the k-Köhler principle. As the exponential impact of the RH was not so pronounced for the PM2.5, it was verified that the best correction is the MLR, considering RH and T.

This work is currently progressing towards extending the field measurements and considering different approaches to calibrate the LCS, such as different algorithms that aim to correct the effect of RH, and machine learning approaches capable of considering several factors that influence the LCS performance. 

The ExpoLIS Sensing System was installed in public buses to monitor AQ in the urban area of Lisbon and test their ability to generate data with high spatial and temporal resolution. The results obtained in the tests performed while in motion indicate that PM concentrations measured inside and outside buses depend on the route and period of the day, and that exposure while commuting can be very high. When expanded to the entire city, this system will inform the citizens about the exposure to air pollutants that results from their choices of daily paths, and even the average estimated dose of air pollutants that they inhale. Furthermore, the Health Optimal Routing System App also informs the citizens about the air pollutant emissions that result from their commuting choices, based on the average emission factors of each transport mode and distance. With this information, we intend to inform and make citizens aware of how their daily commuting choices can improve or deteriorate AQ in urban areas.

In conclusion, the results that are presented in this paper demonstrate the importance of the ExpoLIS App to help citizens to make more informed decisions in order to: (i) reduce their exposure to air pollutants by taking the healthiest path between user-specified departure and arrival locations (not necessarily the shortest one) and (ii) reduce the pollutant emissions by selecting environmental-friendly or public transport modes.

## Figures and Tables

**Table 1:** Bus Routes starting and ending points and hierarchical levels.

Routes (Starting Point-Ending Point)	Bus Route	Hierarchical Level (%)			
		Level 1	Level 2	Level 3	Level 4
Restauradores—Oriente	759	0	10.3	63.6	26.1
Restauradores—Moscavide	744	0	54.8	30.4	14.8
Odivelas—Cais do Sodré	736	5.9	31.2	50.9	12.0
Portas de Benfica—Cais do Sodré	758	0	3.8	91.7	4.5

**Table 2:** Indoor and outdoor PM concentrations (average ± standard deviation) derived I/O ratios for the bus routes analyzed and the range of variation of the meteorological conditions (namely T and RH).

Routes	Starting Time	PM Indoor Concentrations		PM Outdoor Concentrations		I/O		Meteorological Conditions	
		PM2.5 (µg m^−3^)	PM10 (µg m^−3^)	PM2.5 (µg m^−3^)	PM10 (µg m^−3^)	PM2.5	PM10	T (°C)	RH (%)
Restauradores—Oriente	8 a.m.	7.9 ± 2.6	31.0 ± 14.3	5.8 ± 2.0	20.1 ± 10.0	1.4	1.5	23.2–31.2	39.2–57.8
	8 p.m.	(*)	(*)	(*)	(*)	(*)	(*)		
Restauradores—Moscavide	8 a.m.	5.3 ± 2.5	25.1 ± 14.7	2.5 ± 1.2	13.4 ± 11.4	2.1	1.9	22.1–34.3	35.1–62.5
	8 p.m.	5.8 ± 0.7	18.3 ± 7.9	3.3 ± 0.7	10.2 ± 4.6	1.8	1.8		
Odivelas—Cais do Sodré	8 a.m.	8.7 ± 2.6	29.9 ± 14.3	4.5 ± 2.1	15.2 ± 9.9	1.9	2.0	20.7–31.2	35.8–62.0
	8 p.m.	8.8 ± 5.2	42.0 ± 39.3	9.5 ± 5.4	35.9 ± 26.9	0.9	1.2		
Portas de Benfica—Cais do Sodré	8 a.m.	4.9 ± 2.8	21.1 ± 15.6	1.4 ± 0.8	4.2 ± 3.7	3.3	5.0	19.0–28.9	28.4–47.9
	8 p.m.	7.2 ± 2.1	26.5 ± 10.6	2.3 ± 1.0	6.2 ± 4.2	3.1	4.3		

(*) Due to a systematic error in the sensor’s reading the night period measurements were not considered.

**Table 3:** Inhalation rate (m^3^ h^−1^) applied in the ExpoLIS app for each transport mode.

Transport Mode	IR (m^3^ h^−1^)
Bicycle	1.41
Car	0.71
Bus	0.76
Motorcycle	0.94
Walking	1.15

**Table 4:** PM2.5 and PM10 inhaled doses (µg) for each selected route during a two-way trip.

Routes	Starting Time	Travelling Time (min)	PM2.5 Inhaled Doses (µg)				PM10 Inhaled Doses (µg)			
			Bus	Car	Motorcycle	Bicycle	Bus	Car	Motorcycle	Bicycle
Restauradores—Oriente	8 a.m.	125	12.5	3.9	4.9	13.1	49.2	13.0	16.9	45.3
	8 p.m.	-	-	-	-	-	-	-	-	-
Restauradores—Moscavide	8 a.m.	137	9.2	2.6	3.3	8.9	43.7	13.7	17.8	47.9
	8 p.m.	111	8.2	3.5	4.4	11.9	25.8	10.4	13.5	36.4
Odivelas—Cais do Sodré	8 a.m.	145	16.0	3.5	4.4	11.8	55.1	11.4	14.9	40.0
	8 p.m.	121	13.5	7.4	9.3	25.0	64.5	27.0	35.1	94.5
Portas de Benfica—Cais do Sodré	8 a.m.	111	6.9	0.7	0.9	2.4	29.7	2.0	2.6	7.1
	8 p.m.	83	7.6	1.1	1.4	3.9	27.9	3.0	3.9	10.5

**Table 5:** Emission factors of sample motorized transport modes, fuel types, and Euro standards.

Vehicle	Fuel	Euro Standard	Emission Factor (g PM km^−1^)
passenger car	petrol	Euro 5	0.00061
	diesel	Euro 5	0.00025
	electric	–	0
bus	biodiesel	Euro 5	0.03138
	diesel	Euro 5	0.03138
	electric	-	0
	CNG	Euro 5	0.01
motorcycle	petrol	Euro 5	0.00297

**Table 6:** PM emissions (g per passenger) of sample motorized transport modes, fuel types, and Euro standards.

Route	Distance (km)	Emissions (g PM Per Passenger)					
		Passenger Car		Bus			Motorcycle
		Petrol	Diesel	Biodiesel	Diesel	CNG	Petrol
Restauradores—Oriente	24	0.015	0.006	0.01506	0.01506	0.0048	0.071
Restauradores—Moscavide	38	0.023	0.009	0.02384	0.02384	0.0076	0.113
Odivelas—Cais do Sodré	28	0.017	0.007	0.01758	0.01758	0.0056	0.083
Portas de Benfica—Cais do Sodré	18	0.011	0.004	0.0113	0.0.0113	0.0036	0.054

## Data Availability

Data available on request.

## Supplementary Materials

The following supporting information can be downloaded at: https://www.mdpi.com/article/10.3390/ijerph20054073/s1, Figure S1: Correlation between PM10 concentrations measured by the OPC-N3 sensor and RH; Figure S2: Correlation between PM2.5 concentrations measured by the OPC-N3 sensor and RH.
