# Supplementary material for: A Low-Cost Sensor System Installed in Buses to Monitor Air Quality in Cities
Source: Int J Environ Res Public Health. 2023 Feb 24;20(5):4073. doi: 10.3390/ijerph20054073 (PMC10002067; doi:10.3390/ijerph20054073)
Supplement: Supplementary file 1 [file ijerph-20-04073-s001.zip › ijerph-2134959-supplementary.pdf]

Supplementary Materials

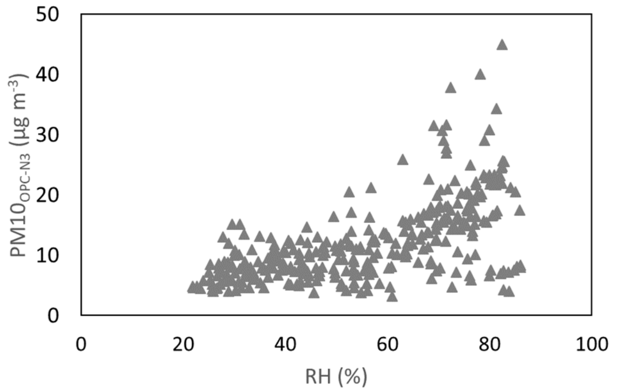

Figure S1. Correlation between PM10 concentrations measured by the OPC-N3 sensor and RH.

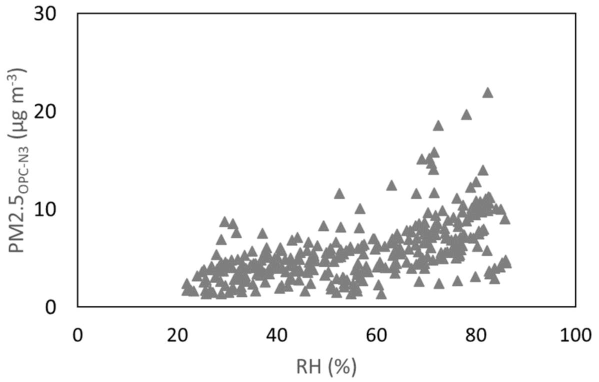

Figure S2. Correlation between PM2.5 concentrations measured by the OPC-N3 sensor and RH.
